# Sex-related molecular phenotypes in anxiety-depressive disorders: a machine learning analysis of routine blood biomarkers

**DOI:** 10.3389/fpsyt.2026.1889453

**Published:** 2026-07-23

**Authors:** Weizhe Zhen, Jingjing Chen, Hongjun Zhen, Yanli Zhang, Shiyu Du, Weihe Zhang, Dantao Peng

**Affiliations:** 1 Graduate School, Capital Medical University, Beijing, China; 2 Graduate School, Beijing University of Chinese Medicine, Beijing, China; 3 Department of Orthopedics, Handan Chinese Medicine Hospital, Handan, Hebei, China; 4 Department of Gastroenterology, China-Japan Friendship Hospital, Beijing, China; 5 Department of Neurology, China-Japan Friendship Hospital, Beijing, China

**Keywords:** anxiety disorders, blood biomarkers, depressive disorders, machine learning, molecular phenotyping, sex differences

## Abstract

**Background:**

Anxiety disorders and depressive disorders are the most prevalent mental disorders worldwide. Their diagnosis has long relied on clinical symptom assessment, and objective blood−based biomarkers remain lacking. Sex is a critical risk factor for these disorders; however, sex−specific divergence in blood biochemical profiles has yet to be systematically characterized.

**Methods:**

This retrospective study enrolled 778 patients diagnosed with anxiety−depressive state at China−Japan Friendship Hospital. Demographic data, complete blood count parameters, and blood biochemical parameters were collected. Following missing value processing and multiple imputation, Mann–Whitney U tests were applied to identify sex−differentially expressed biomarkers. A random forest classifier was constructed to evaluate the discriminative capacity of combined multi−marker panels, with model performance comprehensively assessed through receiver operating characteristic curve analysis, SHAP−based explainability analysis, and multi−classifier probability projection. Age−stratified analyses were performed with a threshold of 50 years to explore the potential modifying effect of age on sex differences.

**Results:**

Several biomarkers exhibiting significant differences between males and females were identified (FDR < 0.05), among which creatinine, hemoglobin, hematocrit, red blood cell count, and uric acid demonstrated the largest effect sizes. The random forest model achieved an area under the receiver operating characteristic curve of 0.902 on the independent test set. Multi−classifier probability projection following hyperparameter tuning yielded a Silhouette coefficient of 0.464 in the two−dimensional space, with permutational multivariate analysis of variance confirming highly significant centroid differences between groups (p < 0.001). Age−stratified analysis using hemoglobin as an example revealed that levels in males were significantly higher than those in females across both age strata, with the magnitude of the sex difference attenuated in the ≥50−year group compared with the <50−year group.

**Conclusions:**

Robust sex−related signals are embedded in routine blood biochemical markers. Although complete separation is difficult to achieve under unsupervised dimensionality reduction, these signals can be efficiently integrated through ensemble learning algorithms. This study provides a molecular phenotypic basis related to sex in patients with anxiety−depressive state and underscores the importance of fully considering sex as a variable in clinical laboratory testing.

## Background

Anxiety disorders and depressive disorders are among the most prevalent mental disorders worldwide (1–3). In the United States, approximately 9% of adults experience major depressive disorder annually, with a lifetime prevalence of approximately 17% in males and 30% in females (4). However, the diagnosis of anxiety and depression has long relied on clinical symptom assessment, lacking objective blood-based biomarkers, which results in low early recognition rates and high misdiagnosis rates (5, 6). In recent years, peripheral blood biomarkers have garnered considerable attention owing to their non-invasive nature and reproducibility. Lipids have demonstrated potential diagnostic utility in major depressive disorder and anxiety disorders. Triglycerides, total cholesterol, low-density lipoprotein cholesterol, and other lipid markers exhibit significant alterations in affected patients; however, further investigation is warranted to delineate disease-specific biomarker profiles (7). Researchers conducted a systematic review and meta-analysis examining neurofilament light chain (NfL) and glial fibrillary acidic protein (GFAP) in mood and anxiety disorders, finding no significant elevation of NfL or GFAP in patients with depression, whereas NfL levels were elevated in bipolar disorder (8). Building on these observations, multi-marker screening strategies grounded in proteomics and metabolomics are progressively being applied to the discovery of biomarkers for psychiatric disorders (9–19). Nevertheless, the biomarkers examined and the analytical platforms employed in these studies are associated with relatively high costs, limiting their accessibility in many primary healthcare settings (16, 20–22).

Sex is a critical risk factor in the epidemiology of anxiety and depression (23–26). Substantial epidemiological evidence demonstrates that the prevalence of anxiety and depression is higher in females than in males (23, 27, 28). A comprehensive umbrella review confirmed that the prevalence of internalizing disorders, including anxiety and depression, is significantly higher in females, whereas externalizing disorders are more prevalent in males (23, 28). Researchers (29) systematically delineated the role of sex in the environmental risk factors, symptom onset, genetic susceptibility, and disease progression of major depressive disorder, generalized anxiety disorder, and post-traumatic stress disorder, highlighting that the elevated prevalence of internalizing disorders in females involves multiple interactions between sex hormones and psychosocial factors. Furthermore, previous study (30) comprehensively examined sex differences in the immune system in the context of mood disorders, reporting that females not only exhibit a higher prevalence of autoimmune diseases but also mount stronger immune responses to stress, suggesting that sex may influence the susceptibility and clinical manifestations of psychiatric disorders through immuno-inflammatory mechanisms.

The influence of sex on blood biomarkers in patients with anxiety and depression represents a prominent focus of precision psychiatry research in recent years. Researchers (9) utilized a multiplex immunoassay platform to quantify 171 serum molecules, identifying pronounced sex-specific associations between major depressive disorder (MDD) and immune response-related proteins: MDD in males was specifically associated with elevations in inflammatory markers including C-reactive protein (CRP), cystatin C, and β2-microglobulin, whereas a comparably broad immune activation pattern was not observed in females. An analysis of 144,890 participants from the UK Biobank demonstrated that the association between CRP concentrations and depressive and anxiety symptoms was more pronounced in females than in males, while Mendelian randomization analyses suggested that altered activity of the IL-6/IL-6R pathway may constitute a risk factor for depression (31). Notably, some researchers (32) reported in the Netherlands Study of Depression and Anxiety (NESDA) that elevated CRP was present only in males with current anxiety disorders, not in females, indicating potential sex-specific heterogeneity in the direction of the association between inflammatory markers and anxiety. Previous study (33) further corroborated that inflammatory, neurotrophic, and serotonergic marker levels are more markedly elevated in females with depression, and that the correlations between these markers and symptom severity are also stronger in females. Collectively, the existing evidence indicates that blood biomarkers of anxiety and depression exhibit pronounced sexual dimorphism, potentially involving immuno-inflammatory, neuroendocrine, and metabolic pathways, thereby underscoring the clinical importance of developing sex-stratified diagnostic and therapeutic strategies.

## Methods

### Data source and preprocessing

This study was a retrospective investigation that enrolled 778 patients diagnosed with anxiety-depressive state at China-Japan Friendship Hospital between June 2023 and December 2024. Demographic information and multiple routine blood biochemical parameters were collected for all participants. All personal identifiers were removed prior to data retrieval. This study was approved by the Institutional Review Board of China–Japan Friendship Hospital (approval No. 2020-31-Y06-32). The requirement for informed consent was waived owing to the retrospective nature of the study.

Biomarker variable names were systematically cleaned by removing Chinese characters and special symbols, and were converted to standardized English format. The sex variable was recoded as a factor variable. All biomarker values were extracted using regular expressions and coerced to numeric type; records that could not be converted were treated as missing values. The proportion of missing data was calculated for each biomarker: variables with a missing rate exceeding 40% were directly excluded, whereas variables with a missing rate ≤40% were imputed using the predictive mean matching (PMM) method within the Multiple Imputation by Chained Equations (MICE) framework, with five iterations, generating a complete dataset for multivariate analysis. For descriptive statistics summarizing the demographic and blood parameters of the enrolled population, no variable exclusion or imputation was performed; descriptive statistics were computed directly from the non-missing values of the original data to preserve complete variable information.

### Descriptive statistics of baseline characteristics

The mean, standard deviation, and 95% confidence interval were calculated for all variables in the overall population as well as in the male and female subgroups, presented in the format “mean ± standard deviation (95% CI lower limit, upper limit).” For comparisons between male and female groups, Welch’s t-test was applied to continuous variables, and the chi-square test or Fisher’s exact test was applied to categorical variables depending on expected frequencies; p < 0.001 was uniformly reported as “< 0.001.”

### Univariate differential analysis

For all low-missingness biomarkers following imputation, the Mann–Whitney U test was employed to compare the distributional differences between males and females. The rank-biserial correlation coefficient was calculated as the effect size. The Benjamini–Hochberg method was applied to adjust the raw p-values for multiple comparisons, yielding the false discovery rate (FDR). A volcano plot was constructed with effect size on the horizontal axis and −log_10_(FDR) on the vertical axis; biomarkers with FDR < 0.05 were defined as significantly sex-differential. The values of these significant biomarkers were subsequently standardized, and a heatmap of the group means was generated to visualize the direction and magnitude of the differences.

### Random forest modeling and feature importance evaluation

The dataset was randomly partitioned into a training set (70%) and an independent test set (30%). A random forest classifier was constructed on the training set (default mtry = √p, ntree = 500), and the predicted probabilities for the female class were generated on the test set. The receiver operating characteristic (ROC) curve was plotted and the area under the curve (AUC) was computed to assess the discriminative performance of the model. Features were ranked by Gini importance (Mean Decrease Gini), and the top 25 variables were visualized. The top four most important variables were further displayed using violin plots to illustrate their distributions in males and females.

### Model hyperparameter tuning and explainability analysis

To further optimize classification performance and provide mechanistic interpretation of model decisions, hyperparameter tuning of the random forest was conducted on the training set. A 5-fold cross-validation procedure was employed to traverse candidate mtry values (ranging from 1 to 2√p) and ntree values (300, 500, 800), and the parameter combination yielding the highest average AUC was selected to retrain the final model. The AUC of the tuned model was evaluated on the independent test set, and the corresponding ROC curve was plotted. The SHAP (Shapley Additive Explanations) method was applied to the random forest model for global feature importance assessment and individual feature dependence analysis, revealing the direction and magnitude of each biomarker’s contribution to the model predictions.

### Multi-classifier probability projection and assessment of sex separation

A projection strategy based on multi-classifier probability scores was adopted to evaluate the capacity of blood biochemical markers to discriminate between sexes and to quantify the degree of separation on the independent test set. Following missing data handling, the dataset was randomly divided into a training set and an independent test set at a 7:3 ratio. Random forest and support vector machine (SVM) classifiers were separately constructed on the training set. Hyperparameter tuning was performed using 5-fold cross-validation grid search: for random forest, the mtry and ntree parameters were optimized; for SVM, the cost and γ parameters of the radial basis kernel were optimized; in each case, the objective was to maximize the area under the ROC curve (AUC). The tuned models were then used to generate predicted male probabilities for the test set samples, forming a two-dimensional classifier score space. The Euclidean distance was computed in this space, from which the Silhouette coefficient was derived to quantify the degree of separation between the male and female groups. Permutational multivariate analysis of variance (PERMANOVA, 999 permutations) was employed to test the significance of the group centroid difference. If the Silhouette coefficient in the two-dimensional projection was below 0.5, extreme gradient boosting (XGBoost) was introduced as a third classifier to construct a three-dimensional score space, and the separation was re-evaluated. The projection dimensionality yielding the highest Silhouette coefficient was ultimately selected for visualization, with the corresponding Silhouette coefficient and PERMANOVA p-value reported.

### Age-stratified analysis

To investigate the potential modifying effect of age on sex differences, the study population was stratified by the age of 50 years into a younger group (<50 years) and an older group (≥50 years). Among the top four biomarkers identified by random forest importance, the one that was empirically present in the dataset and exhibited a significant sex difference (the second-ranking biomarker was preferentially selected) was chosen. Within each age stratum, boxplots were used to compare the distributions between males and females, with the Wilcoxon test p-value annotated.

### Software

All statistical analyses and figure generation were performed using R version 4.5.3.

## Results

### Baseline characteristics of the study population

**Figure 1:**
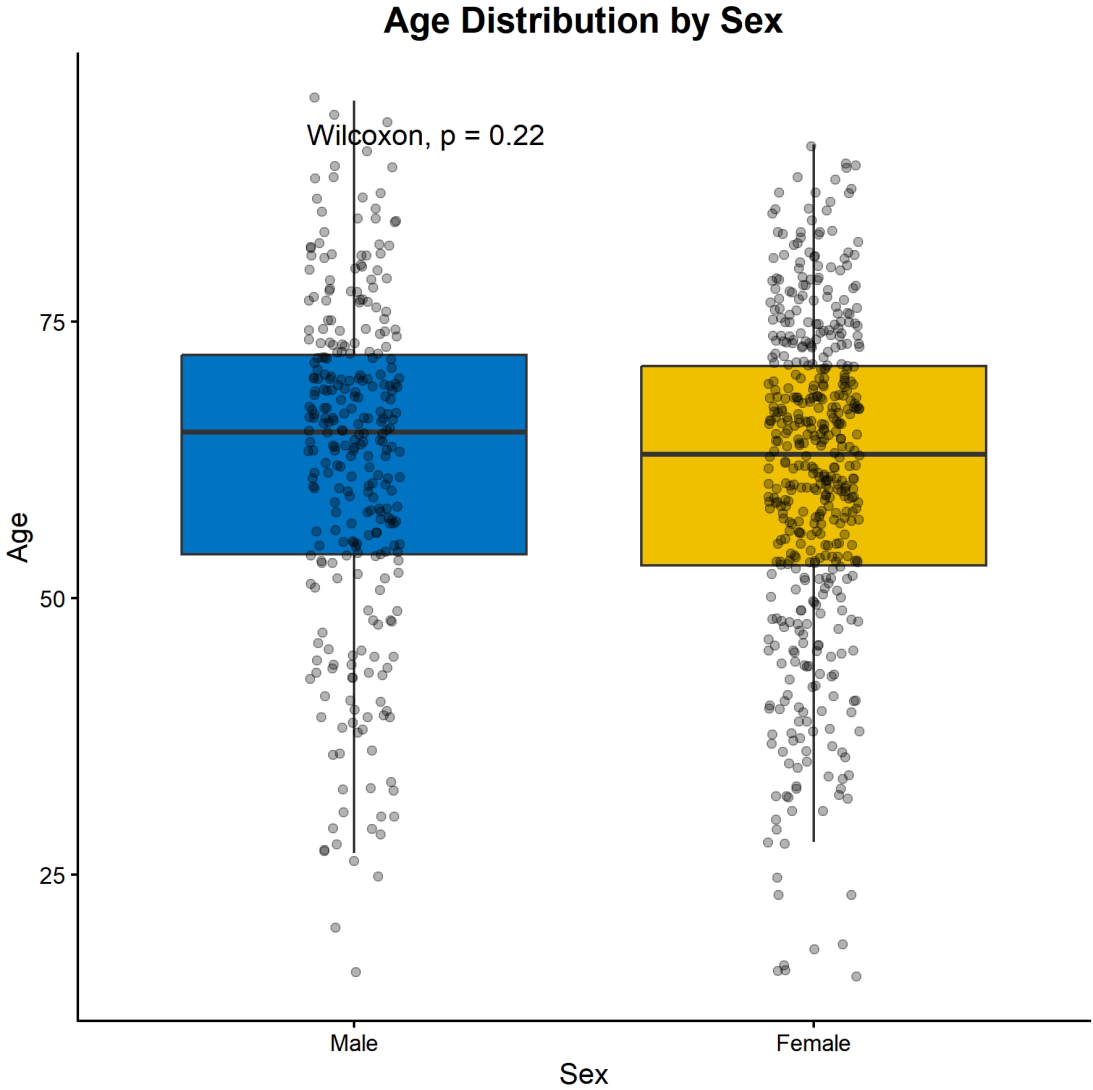
Age distribution of the study population. Boxplots showing the age distribution of male and female participants. Each dot represents an individual. The horizontal line inside the box indicates the median, and the lower and upper hinges correspond to the first and third quartiles. The p value was derived from the Wilcoxon rank-sum test.

A total of 778 eligible patients were enrolled in this study. The mean, standard deviation, 95% confidence interval, and p-value for all continuous variables in the overall population and by sex subgroup are presented in **Table 1**. No significant difference in age distribution was observed between males and females (**Figure 1**).

**Table 1:** Characteristics of the patients.

Variable	Overall (N = 778)	Male (N = 294)	Female (N = 484)	P-value*
Demographics				
Age (years)	61.54 ± 14.68 (60.51, 62.58)	62.30 ± 14.99 (60.59, 64.01)	61.08 ± 14.49 (59.79, 62.38)	0.22
Glycemic and lipid profile				
GLU (mmol/L)	6.31 ± 2.42 (6.14, 6.48)	6.63 ± 2.62 (6.33, 6.93)	6.11 ± 2.27 (5.91, 6.31)	0.005
TG (mmol/L)	1.61 ± 1.38 (1.50, 1.72)	1.65 ± 1.82 (1.43, 1.87)	1.58 ± 0.98 (1.48, 1.68)	0.593
CHO-TC (mmol/L)	4.51 ± 1.71 (4.37, 4.64)	4.20 ± 1.65 (3.99, 4.40)	4.71 ± 1.72 (4.54, 4.88)	<0.001
HDL-C (mmol/L)	1.25 ± 0.35 (1.22, 1.27)	1.13 ± 0.30 (1.10, 1.17)	1.32 ± 0.36 (1.29, 1.35)	<0.001
LDL-C (mmol/L)	2.73 ± 1.26 (2.63, 2.83)	2.60 ± 1.63 (2.40, 2.80)	2.81 ± 0.93 (2.72, 2.91)	0.055
Liver and kidney function				
ALT (U/L)	25.94 ± 25.16 (24.16, 27.71)	28.51 ± 28.17 (25.29, 31.74)	24.37 ± 23.01 (22.31, 26.42)	0.034
AST (U/L)	25.55 ± 36.05 (23.01, 28.09)	26.32 ± 40.66 (21.65, 30.98)	25.08 ± 32.98 (22.14, 28.03)	0.662
TBIL (μmol/L)	14.16 ± 20.44 (12.72, 15.60)	14.62 ± 6.92 (13.83, 15.42)	13.88 ± 25.34 (11.62, 16.15)	0.548
DBIL (μmol/L)	3.19 ± 18.13 (1.91, 4.47)	2.97 ± 2.51 (2.68, 3.26)	3.33 ± 22.90 (1.28, 5.38)	0.734
ALB (g/L)	41.13 ± 4.22 (40.77, 41.49)	41.54 ± 4.47 (40.94, 42.15)	40.86 ± 4.05 (40.43, 41.30)	0.077
TBA (μmol/L)	5.34 ± 14.75 (4.28, 6.39)	5.48 ± 8.23 (4.53, 6.44)	5.25 ± 17.58 (3.66, 6.84)	0.802
MAO (U/L)	5.90 ± 3.82 (5.58, 6.22)	5.83 ± 3.10 (5.40, 6.26)	5.94 ± 4.19 (5.50, 6.38)	0.730
CK (U/L)	107.39 ± 290.59 (81.34, 133.44)	125.51 ± 347.71 (77.20, 173.82)	94.47 ± 241.61 (66.12, 122.82)	0.278
Urea (mmol/L)	5.89 ± 3.86 (5.62, 6.16)	6.31 ± 4.19 (5.83, 6.79)	5.63 ± 3.63 (5.30, 5.96)	0.021
CR (μmol/L)	75.98 ± 94.04 (69.36, 82.61)	88.43 ± 111.40 (75.65, 101.20)	68.44 ± 80.95 (61.21, 75.67)	0.008
eGFR (mL/min/1.73m²)	92.22 ± 22.61 (90.62, 93.81)	91.59 ± 21.38 (89.14, 94.05)	92.60 ± 23.34 (90.51, 94.68)	0.542
UA (μmol/L)	314.60 ± 97.00 (307.74, 321.47)	348.71 ± 97.67 (337.47, 359.95)	293.87 ± 90.62 (285.74, 302.00)	<0.001
Electrolytes and minerals				
CO_2_ (mmol/L)	25.42 ± 2.86 (25.22, 25.63)	25.05 ± 2.93 (24.72, 25.39)	25.65 ± 2.80 (25.40, 25.90)	0.006
K (mmol/L)	4.01 ± 1.04 (3.94, 4.08)	4.00 ± 0.42 (3.95, 4.05)	4.02 ± 1.27 (3.90, 4.13)	0.811
Na (mmol/L)	140.00 ± 11.31 (139.20, 140.79)	139.16 ± 3.36 (138.77, 139.54)	140.51 ± 14.07 (139.25, 141.76)	0.044
Cl (mmol/L)	104.88 ± 6.35 (104.44, 105.33)	103.84 ± 9.27 (102.77, 104.90)	105.52 ± 3.43 (105.21, 105.82)	0.003
Ca (mmol/L)	2.28 ± 0.12 (2.27, 2.28)	2.27 ± 0.12 (2.26, 2.29)	2.28 ± 0.12 (2.27, 2.29)	0.641
IP (mmol/L)	1.18 ± 0.23 (1.16, 1.19)	1.13 ± 0.22 (1.11, 1.16)	1.20 ± 0.23 (1.18, 1.22)	<0.001
Complete blood count				
WBC (×10^9^/L)	6.59 ± 2.67 (6.40, 6.78)	7.26 ± 2.96 (6.92, 7.60)	6.18 ± 2.38 (5.97, 6.39)	<0.001
NEUT (×10^9^/L)	4.29 ± 2.54 (4.11, 4.47)	4.87 ± 2.88 (4.54, 5.20)	3.94 ± 2.23 (3.74, 4.14)	<0.001
LYMPH (×10^9^/L)	1.72 ± 0.70 (1.68, 1.77)	1.73 ± 0.73 (1.65, 1.82)	1.72 ± 0.67 (1.66, 1.78)	0.799
MONO (×10^9^/L)	0.41 ± 0.26 (0.39, 0.43)	0.46 ± 0.19 (0.44, 0.48)	0.39 ± 0.29 (0.36, 0.41)	<0.001
EO (×10^9^/L)	0.15 ± 0.26 (0.13, 0.16)	0.17 ± 0.24 (0.14, 0.19)	0.13 ± 0.28 (0.11, 0.16)	0.069
BASO (×10^9^/L)	0.04 ± 0.22 (0.02, 0.06)	0.04 ± 0.06 (0.03, 0.04)	0.04 ± 0.27 (0.02, 0.07)	0.711
NEUT (%)	62.36 ± 12.34 (61.49, 63.23)	64.35 ± 12.07 (62.97, 65.74)	61.15 ± 12.36 (60.04, 62.25)	<0.001
LYM (%)	28.45 ± 10.83 (27.69, 29.22)	26.11 ± 10.39 (24.91, 27.30)	29.88 ± 10.85 (28.91, 30.85)	<0.001
MONO (%)	6.43 ± 2.48 (6.26, 6.61)	6.57 ± 2.21 (6.31, 6.82)	6.35 ± 2.63 (6.11, 6.58)	0.214
EO (%)	2.19 ± 1.92 (2.06, 2.33)	2.39 ± 2.23 (2.13, 2.64)	2.07 ± 1.69 (1.92, 2.22)	0.037
BASO (%)	0.49 ± 0.30 (0.47, 0.51)	0.51 ± 0.31 (0.47, 0.54)	0.48 ± 0.30 (0.46, 0.51)	0.270
RBC (×10¹²/L)	4.24 ± 0.62 (4.19, 4.28)	4.48 ± 0.67 (4.40, 4.55)	4.09 ± 0.54 (4.04, 4.14)	<0.001
HGB (g/L)	128.19 ± 18.43 (126.89, 129.49)	136.41 ± 19.62 (134.16, 138.66)	123.18 ± 15.69 (121.78, 124.59)	<0.001
HCT (%)	38.00 ± 6.06 (37.58, 38.43)	39.95 ± 5.41 (39.33, 40.57)	36.82 ± 6.14 (36.27, 37.37)	<0.001
MCV (fL)	89.55 ± 5.72 (89.15, 89.95)	89.36 ± 5.77 (88.70, 90.02)	89.66 ± 5.69 (89.15, 90.17)	0.477
MCH (pg)	30.31 ± 2.26 (30.15, 30.47)	30.55 ± 1.94 (30.33, 30.77)	30.16 ± 2.42 (29.95, 30.38)	0.015
MCHC (g/L)	338.29 ± 11.70 (337.47, 339.12)	341.38 ± 12.12 (339.99, 342.77)	336.41 ± 11.02 (335.43, 337.40)	<0.001
RDW-SD (fL)	42.40 ± 4.72 (42.06, 42.73)	42.12 ± 4.66 (41.59, 42.66)	42.56 ± 4.75 (42.14, 42.99)	0.208
RDW-CV (%)	13.00 ± 1.40 (12.90, 13.10)	12.91 ± 1.19 (12.78, 13.05)	13.06 ± 1.52 (12.92, 13.19)	0.152
PLT (×10^9^/L)	221.92 ± 73.92 (216.71, 227.14)	213.11 ± 74.65 (204.55, 221.67)	227.28 ± 73.03 (220.75, 233.82)	0.010
PDW (fL)	11.59 ± 4.14 (11.30, 11.88)	11.43 ± 2.02 (11.20, 11.66)	11.68 ± 5.01 (11.23, 12.13)	0.328
MPV (fL)	10.26 ± 0.89 (10.20, 10.32)	10.20 ± 0.90 (10.10, 10.31)	10.29 ± 0.88 (10.21, 10.37)	0.192
P-LCR (%)	26.76 ± 8.38 (26.16, 27.35)	26.57 ± 10.04 (25.41, 27.72)	26.87 ± 7.18 (26.23, 27.52)	0.647
PCT (%)	0.22 ± 0.06 (0.22, 0.23)	0.21 ± 0.06 (0.21, 0.22)	0.23 ± 0.06 (0.22, 0.24)	<0.001
Coagulation and cardiac markers				
PT (s)	13.23 ± 1.39 (13.13, 13.33)	13.46 ± 1.69 (13.27, 13.66)	13.09 ± 1.15 (12.98, 13.19)	<0.001
PTA (%)	100.21 ± 14.93 (99.15, 101.28)	97.42 ± 16.22 (95.54, 99.30)	101.93 ± 13.82 (100.67, 103.18)	<0.001
INR	1.02 ± 0.14 (1.01, 1.03)	1.04 ± 0.17 (1.02, 1.06)	1.00 ± 0.11 (0.99, 1.01)	0.001
APTT (s)	35.45 ± 10.31 (34.72, 36.19)	36.72 ± 15.20 (34.95, 38.48)	34.68 ± 5.37 (34.19, 35.17)	0.030
Fib (g/L)	3.52 ± 1.04 (3.45, 3.60)	3.47 ± 1.06 (3.35, 3.59)	3.55 ± 1.04 (3.46, 3.65)	0.314
TT (s)	17.07 ± 3.84 (16.79, 17.34)	16.94 ± 1.31 (16.79, 17.09)	17.14 ± 4.77 (16.71, 17.58)	0.384
D-D (mg/L)	0.85 ± 2.05 (0.70, 1.00)	0.79 ± 1.89 (0.57, 1.01)	0.89 ± 2.15 (0.69, 1.09)	0.526
FDP (mg/L)	4.16 ± 10.24 (3.26, 5.05)	4.38 ± 10.34 (2.92, 5.84)	4.01 ± 10.19 (2.87, 5.15)	0.695
hsTnI (ng/mL)	0.03 ± 0.40 (0.00, 0.06)	0.05 ± 0.57 (-0.02, 0.12)	0.02 ± 0.23 (-0.00, 0.04)	0.442
CK-MB (U/L)	2.91 ± 18.13 (1.50, 4.31)	3.68 ± 22.82 (0.88, 6.49)	2.40 ± 14.26 (0.98, 3.82)	0.423
Myo (μg/L)	45.55 ± 107.07 (37.27, 53.82)	46.70 ± 80.15 (36.85, 56.56)	44.79 ± 121.57 (32.71, 56.87)	0.810
BNP (pg/mL)	203.45 ± 593.02 (157.62, 249.29)	216.71 ± 632.64 (138.76, 294.67)	194.85 ± 566.51 (138.63, 251.08)	0.656

GLU, glucose; TG, triglycerides; CHO.TC, total cholesterol; HDL.C, high-density lipoprotein cholesterol; LDL.C, low-density lipoprotein cholesterol; ALT, alanine aminotransferase; AST, aspartate aminotransferase; TBIL, total bilirubin; DBIL, direct bilirubin; ALB, albumin; TBA, total bile acids; MAO, monoamine oxidase; CK, creatine kinase; CR, creatinine; eGFR, estimated glomerular filtration rate; UA, uric acid; CO_2_, carbon dioxide; K, potassium; Na, sodium; Cl, chloride; Ca, calcium; IP, inorganic phosphorus; WBC, white blood cells; NEUT, neutrophils; LYMPH, lymphocytes; MONO, monocytes; EO, eosinophils; BASO, basophils; RBC, red blood cells; HGB, hemoglobin; HCT, hematocrit; MCV, mean corpuscular volume; MCH, mean corpuscular hemoglobin; MCHC, mean corpuscular hemoglobin concentration; RDW, red cell distribution width; PLT, platelets; PDW, platelet distribution width; MPV, mean platelet volume; P-LCR, platelet large cell ratio; PCT, plateletcrit; PT, prothrombin time; PTA, prothrombin activity; INR, international normalized ratio; APTT, activated partial thromboplastin time; Fib, fibrinogen; TT, thrombin time; D-D, D-dimer; FDP, fibrin degradation products; hsTnI, high-sensitivity troponin I; CK-MB, creatine kinase myocardial band; Myo, myoglobin; BNP, brain natriuretic peptide.

### Identification of sex-differentially expressed biomarkers

**Figure 2:**
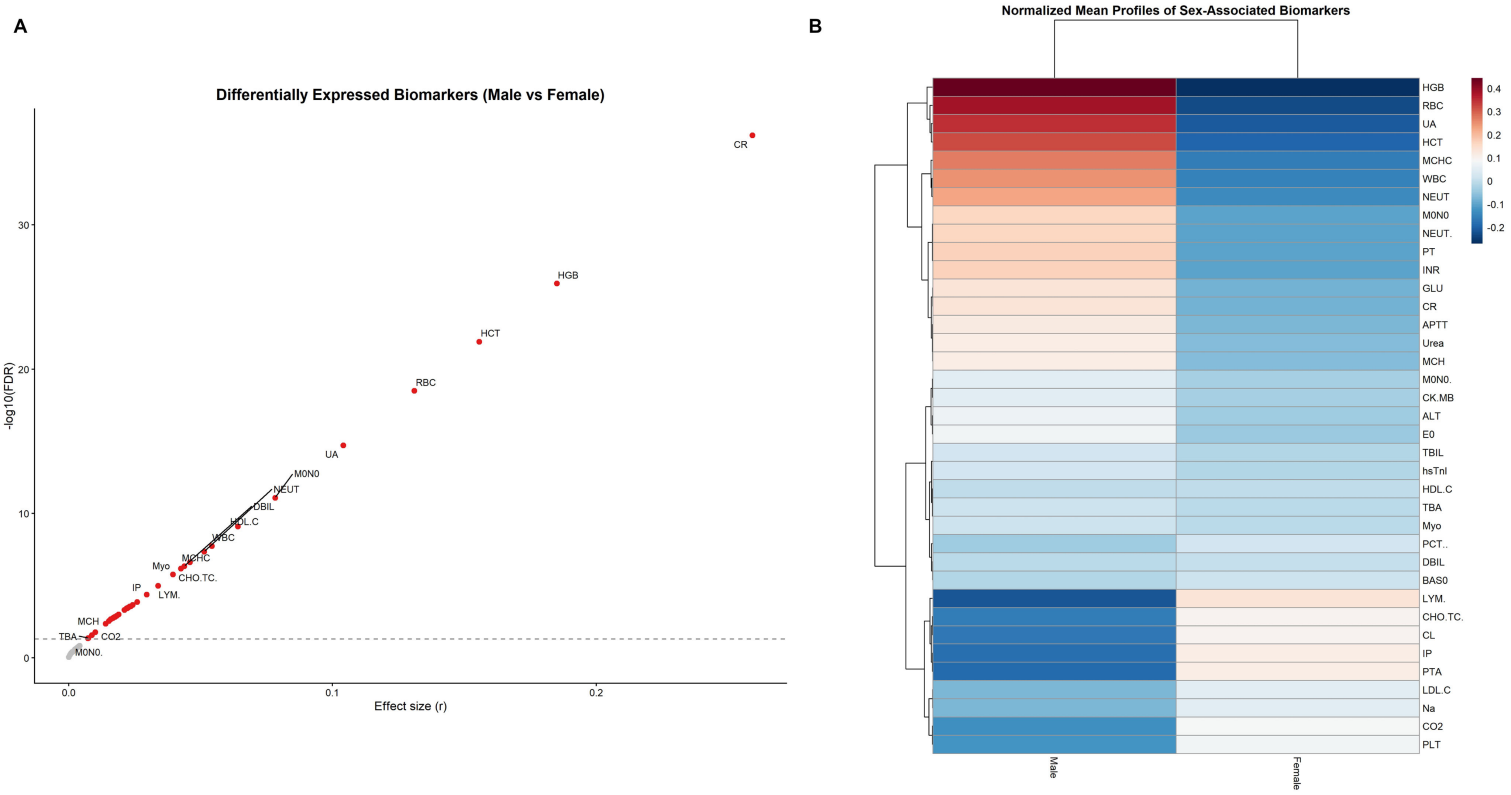
Differentially expressed blood biomarkers between males and females. **(A)** Volcano plot displaying the effect size (rank-biserial correlation coefficient r) versus −log_10_(FDR) for all low-missingness biomarkers. Red points represent biomarkers with FDR < 0.05; gray points indicate non-significant markers. The dashed horizontal line marks the threshold of FDR = 0.05. **(B)** Heatmap of standardized mean values of the significant biomarkers in males and females. Red indicates relatively higher expression and blue indicates relatively lower expression. Rows represent biomarkers and columns represent sex groups. FDR, false discovery rate.

Among all low-missingness biomarkers after imputation, several markers exhibiting significant differences between males and females were identified through Mann–Whitney U testing with FDR correction (FDR < 0.05). The volcano plot intuitively displays the effect size and significance level of each biomarker (**Figure 2A**), with creatinine (Cr), hemoglobin (HGB), hematocrit (HCT), red blood cell count (RBC), and uric acid (UA) showing the largest effect sizes. A heatmap of the standardized values of these significant biomarkers (**Figure 2B**) demonstrates that HGB, RBC, UA, HCT, and related markers are markedly higher in males, whereas Lymphocyte (LYM), Prothrombin Time Activity (PTA) and related markers are higher in females. The standardized mean values of these biomarkers in males and females exhibit opposite directional patterns, and the hierarchical clustering clearly reflects the separation between the sexes.

### Multivariate separation and discriminative performance of the random forest model

**Figure 3:**
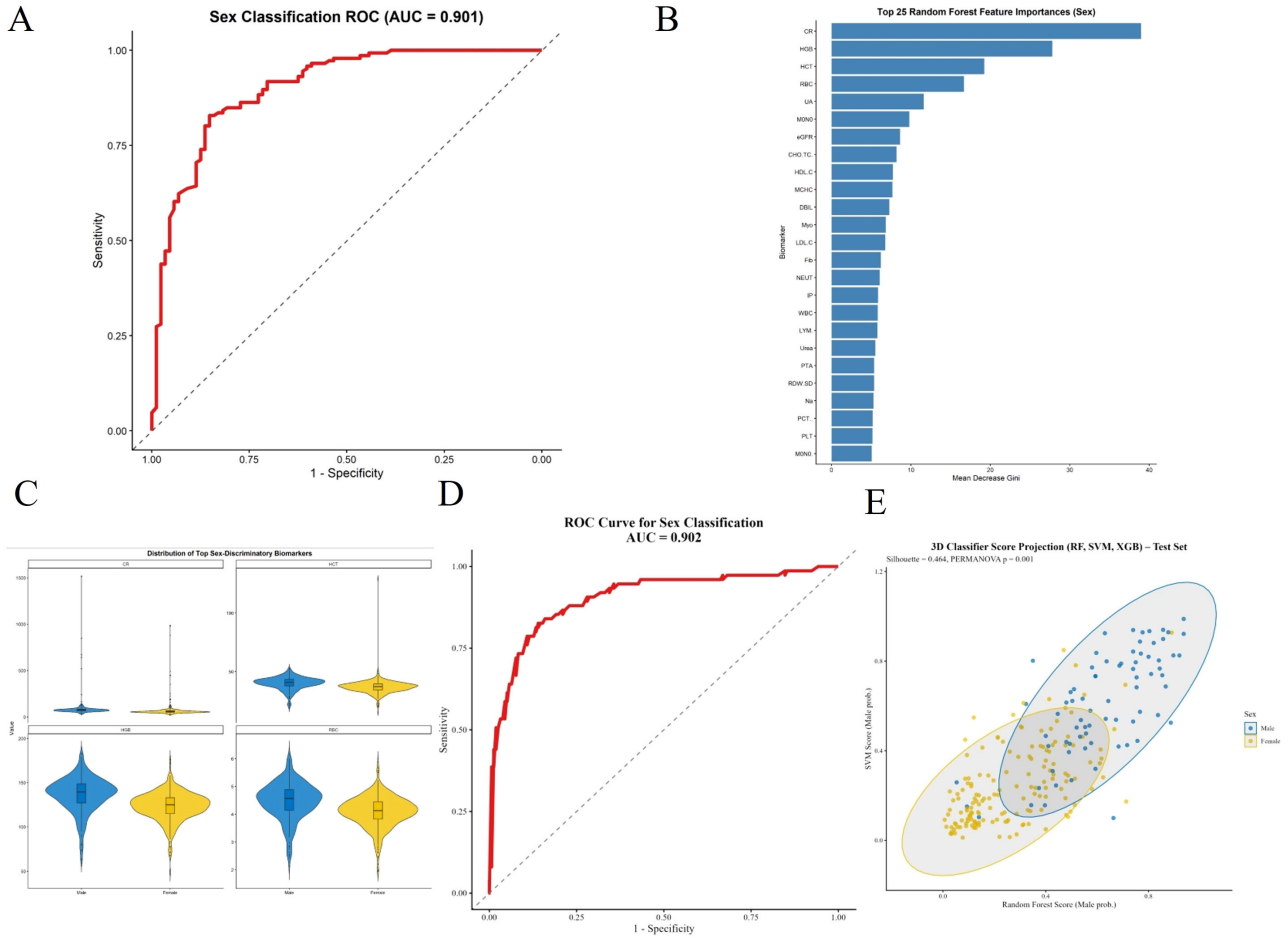
Multivariate modeling and sex classification performance. **(A)** Receiver operating characteristic (ROC) curve of the random forest model evaluated on the independent test set, with an area under the curve (AUC) of 0.901. **(B)** Top 25 variables ranked by random forest feature importance, measured as mean decrease in Gini impurity. **(C)** Violin plots of the top four most important biomarkers identified by random forest, stratified by sex. Embedded boxplots indicate median and interquartile range. **(D)** ROC curve of the hyperparameter-tuned random forest model on the independent test set. **(E)** Two-dimensional classifier score projection constructed from the predicted male probabilities of the tuned random forest and support vector machine (SVM). Each point represents one sample in the test set, and ellipses represent the 95% confidence regions. The reported Silhouette coefficient and permutational multivariate analysis of variance (PERMANOVA) p value were calculated in the original multi-dimensional score space. ROC, receiver operating characteristic; AUC, area under the curve; RF, random forest; SVM, support vector machine; PERMANOVA, permutational multivariate analysis of variance.

On the independent test set, the untuned random forest model achieved an AUC of 0.901 (**Figure 3A**). Feature importance ranking (**Figure 3B**) revealed that Cr, HGB, HCT, and RBC are the most discriminative variables, a finding highly consistent with the univariate analysis results. Violin plots of the top four most important biomarkers (**Figure 3C**) further illustrate their distributional differences between the two groups.

**Figure 4:**
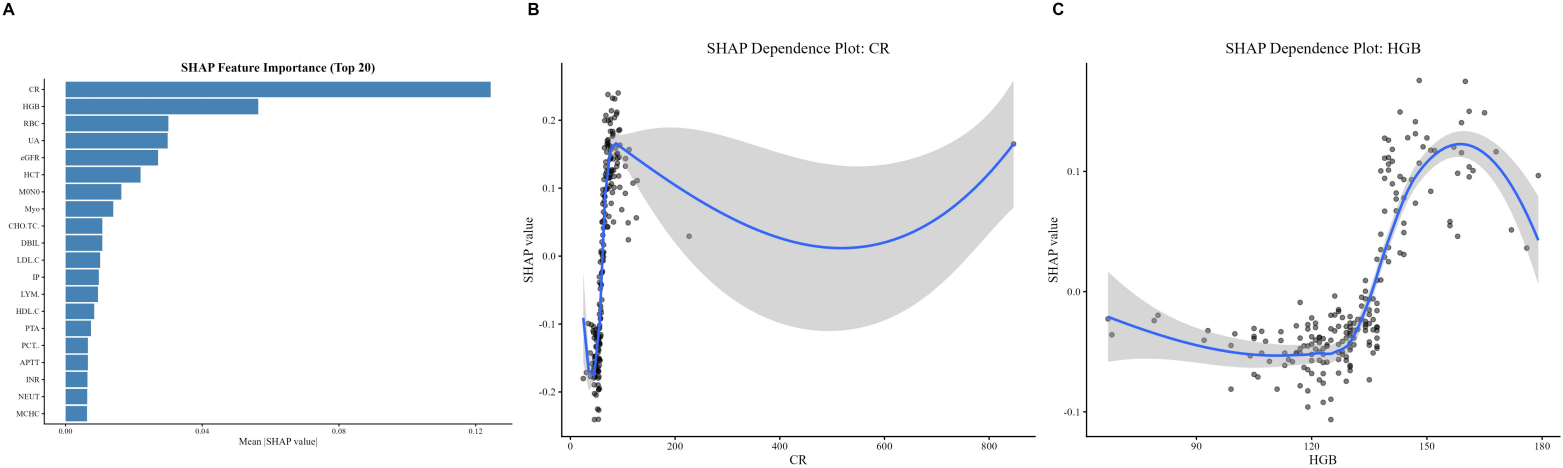
SHAP-based interpretation of the random forest model. **(A)** SHAP feature importance plot showing the top 20 biomarkers with the highest mean absolute SHAP values. The horizontal axis represents the average impact on model output magnitude. **(B)** SHAP dependence plot for creatinine (CR). The x-axis shows the actual CR value, and the y-axis shows the SHAP value. Positive SHAP values push the prediction toward “Male”, while negative values push toward “Female”. The solid curve is a LOESS smoother with a 95% confidence band. **(C)** SHAP dependence plot for hemoglobin (HGB), interpreted as in **(B)**. SHAP, SHapley Additive exPlanations; CR, creatinine; HGB, hemoglobin.

Following hyperparameter tuning, the AUC of the random forest on the test set was further improved to 0.902 (**Figure 3D**). SHAP feature importance analysis confirmed that traditional sexually dimorphic indicators—Cr, UA, and HGB—constitute the core driving forces of the model predictions, and the SHAP dependence plots reveal the monotonic or non-linear relationships between these biomarkers and the predicted probabilities (**Figure 4**).

### Multi-classifier probability projection reveals limited but significant sex separation

After hyperparameter optimization, both random forest and SVM achieved cross-validated AUCs exceeding 0.95 on the training set. In the two-dimensional space constructed from the male probabilities generated by the two classifiers on the independent test set, male and female samples exhibited a discernible separation trend, with a Silhouette coefficient of 0.464; PERMANOVA testing indicated that the difference in multivariate centroids between the two groups was highly significant (p < 0.001). The incorporation of XGBoost to form a three-dimensional probability space did not yield further improvement in the Silhouette coefficient, which remained at a comparable level (**Figure 3E**). These results suggest that, although the sex-related signals embedded in routine blood biochemical markers cannot be fully separated under unsupervised dimensionality reduction, partial separation can be achieved on the test set through multi-classifier probability projection.

### Modifying effect of age on sex differences

**Figure 5:**
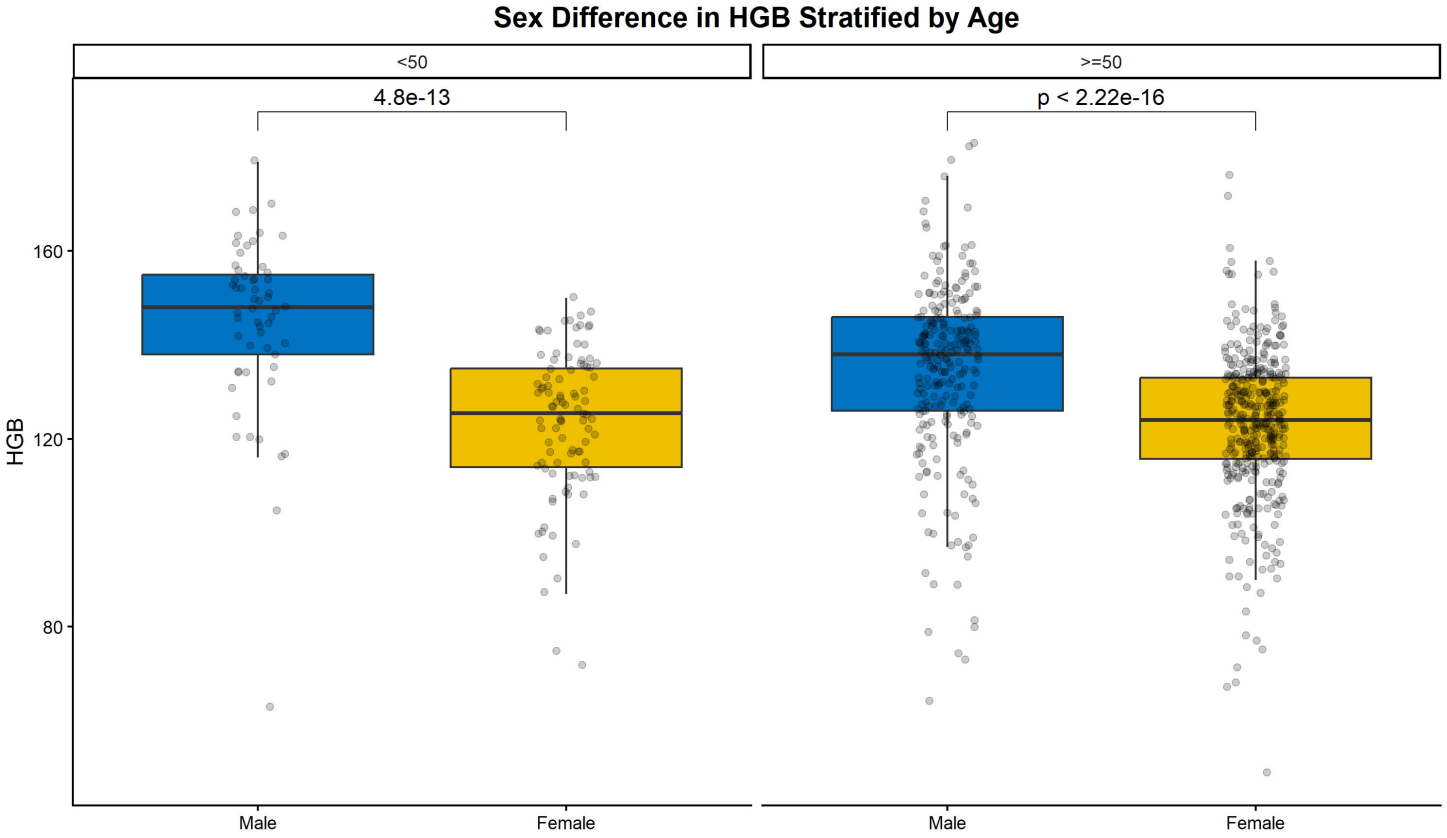
Age-stratified sex comparison of hemoglobin (HGB). The study population was divided into two age groups (<50 years and ≥50 years). Boxplots display HGB levels for males and females within each age stratum. The horizontal line within each box denotes the median, and dots represent individual measurements (jittered horizontally to avoid overlap). Comparisons between males and females within each panel were performed using the Wilcoxon test, with p values.

Taking HGB, a top-ranking biomarker in the random forest importance analysis, as an example, age-stratified analysis (**Figure 5**) demonstrated that HGB levels in males were significantly higher than those in females in both the <50-year and ≥50-year groups. However, the magnitude of the sex difference varied between the two groups: the sex difference was more pronounced in the <50-year group, whereas in the ≥50-year group, female HGB levels exhibited an increase, resulting in a relative narrowing of the sex gap. This trend may reflect the regulatory effect of postmenopausal hormonal changes on relevant biochemical parameters.

## Discussion

Based on the routine blood and biochemical test results of patients with anxiety-depressive state, this study systematically characterized the differential profile of routine blood biochemical markers between male and female patients. Through univariate analysis, machine learning modeling, and multi-classifier probability projection, we quantified the capacity of combined multi-marker panels to discriminate between the sexes, demonstrating significant sex-specific divergence in blood biomarker profiles. These findings provide clinical evidence and a foundation for further investigations into the role of sex in the mechanisms underlying anxiety and depression. Through univariate screening, Cr, HGB, HCT, RBC, and UA were identified as the biomarkers with the largest sex-specific effect sizes, and the standardized heatmap revealed opposite directional patterns in males and females for these markers. Notably, the effect size analysis indicated that, although the sex-specific effect of any individual biomarker is limited, the combination of multiple biomarkers provides substantial complementary information, thereby laying the groundwork for high-accuracy classification. This observation is consistent with the previous findings (34), that employed routine blood biomarkers combined with derived indicators for sex-stratified modeling, further supporting the importance and practical necessity of adopting sex-specific reference intervals in clinical laboratory practice. Recent research has increasingly identified important biomarkers linked to symptom worsening in anxiety and depressive disorders (35). This insight suggests that future work should focus on a secondary screening of the sex-distinguishing biomarkers we identified, to clarify whether any of them drive symptom exacerbation and disease progression in patients of different sexes. It should be emphasized that, within the context of the allostatic load framework (not just limited to a single, distinct biomarker context, but comprehensive burden), we should further discuss how observed patterns of sex-related biomarkers influence different levels of anxiety and depression in men and women. Future research could develop sex-specific composite allostatic load indices to deepen understanding of sexual dimorphism in mood disorders.

With respect to machine learning modeling, the random forest model achieved an AUC exceeding 0.9 on the independent test set, and SHAP analysis identified Cr, UA, and HGB as the core variables driving classification, a finding highly consistent with the univariate analysis. However, the multi-classifier probability projection following hyperparameter tuning yielded a Silhouette coefficient of only 0.464, indicating that male and female samples still exhibit overlap in the two-dimensional probability space. This finding reveals a key characteristic of the sex-related signal in blood biochemistry: although the random forest model can achieve near-perfect separation (AUC > 0.9), complete separation remains difficult to attain when constrained to a low-dimensional probability space. This study further employed SHAP analysis to provide interpretability for the model decisions, demonstrating the value of machine learning explainability in biomarker research. The use of SHAP analysis to uncover differential contribution patterns of routine laboratory markers has similarly become an important methodological approach in current research (36).

Concerning the age-modifying effect, this study found that the sex difference in HGB was more pronounced in the <50-year group, whereas it narrowed in the ≥50-year group due to increased levels in females. This trend can be explained by the regulatory effects of sex hormones on hematological parameters. Estrogen has been demonstrated to influence hematopoiesis and metabolism through multiple pathways (37–43). Estradiol strongly promotes hematopoietic progenitor/stem cell frequency through its action on stromal cells, accompanied by enhanced expression of cell adhesion molecules. Estradiol treatment also enhances the retention of hematopoietic stem cells within the bone marrow vascular niche (36). With regard to metabolism, estrogen lowers serum uric acid levels by stimulating renal urate excretion, exerting a protective effect in premenopausal females. Other Researchers (44) also reported that the genetic effects on LDL-C and UA exhibit sex-interaction effects in females under 50 years of age, attributable to the attenuating effect of estrogen. The postmenopausal increase in HGB in females may reflect the release of estrogen-mediated inhibition of erythropoiesis. This finding suggests that the potential influence of life stage should be considered when interpreting sex differences in clinical settings.

This study has several limitations. First, causal relationships between sex and blood biomarkers were not inferred. Second, the data were derived from a single-center patient population, and the generalizability of the findings requires validation in more diverse patient cohorts. Third, the Silhouette coefficient of 0.464 reflects considerable residual overlap in the test set, which may be associated with an imbalanced sex distribution in the sample or unmeasured confounding factors. Future studies should incorporate multicenter data and explore more refined modeling strategies to further improve sex separation performance. Furthermore, the lack of data from healthy controls inevitably limited our study. This limitation arose because we could not determine whether sex differences in patients with anxiety and depressive disorders differ from those in healthy individuals. Although we did not directly compare the patient group with healthy controls, the biomarkers characterizing sex differences within the anxiety and depressive disorder population remain meaningful. These markers not only exhibit universal sex differences but are also modulated by the disorders themselves. A Korean study found that patients with anxiety and depressive disorders had lower HCT levels than healthy controls (45). Among women, patients showed higher Cr levels and lower RBC counts relative to healthy controls (45). Uric acid levels were also significantly lower in patients than in healthy controls (46). Similarly, recent research has provided evidence for a close link between hemoglobin and anxiety and depressive disorders (47). These observations further support the practical value of using the above blood measures as sex difference biomarkers in individuals with anxiety and depressive disorders.

In conclusion, through multivariate analysis and machine learning, this study reveals the robust sex-related signals embedded in routine blood biochemical markers and demonstrates that, although these signals exhibit limited effect sizes in univariate analyses and are difficult to visualize under unsupervised linear dimensionality reduction, they can be efficiently integrated through ensemble learning algorithms. These findings not only provide a molecular phenotypic basis related to sex in the population of patients with anxiety-depressive state, but also underscore the importance of fully considering sex as a variable in clinical laboratory testing and biomarker research.

## Data Availability

The raw data supporting the conclusions of this article will be made available by the authors, without undue reservation.
